# Electroacupuncture May Alleviate Depression and Exerts Anti‐Inflammatory Effects Through IDO1 Modulation of the Kynurenine Pathway

**DOI:** 10.1155/np/6435038

**Published:** 2026-11-01

**Authors:** Bowen Chen, Xingying Wu, Xihan Ying, Jieqi Zhang, Shuo jiang, Zhong Di, Yi Chen, Yi Huang, Hongen Chang, Ruijie Ma, Qin Guo

**Affiliations:** ^1^ The Third School of Clinical Medicine (School of Rehabilitation Medicine) Key Laboratory of Acupuncture and Neurology of Zhejiang Province Zhejiang Chinese Medical University Hangzhou 310053 Zhejiang China zcmu.edu.cn; ^2^ Department of Neurobiology and Department of Neurology of the Second Affiliated Hospital Zhejiang University School of Medicine Hangzhou 310058 China zju.edu.cn; ^3^ Department of Acupuncture and Moxibustion Zhejiang Provincial Hospital of Chinese Medicine The First Affiliated Hospital of Zhejiang Chinese Medical University Hangzhou 310006 China zjhtcm.com; ^4^ Department of Acupuncture and Moxibustion Third Affiliated Hospital of Zhejiang Chinese Medical University Hangzhou 310009 Zhejiang China zjztyy.com

**Keywords:** depression, electroacupuncture, IDO1, inflammation, kynurenine pathway

## Abstract

**Background:**

Depression is one of the most common mental health conditions worldwide. Numerous studies have identified various factors associated with the onset of depression. Among these factors, inflammation is a key contributor. During the inflammatory process, pro‐inflammatory cytokines activate indole‐2,3‐dioxygenase (IDO), which converts tryptophan (TRP) to kynurenine (KYN) via the KYN pathway (KP). This increases the production of neurotoxic byproducts, thereby exacerbating depressive symptoms. Studies have shown that electroacupuncture (EA) has anti‐inflammatory and antidepressant properties. The present study aimed to investigate the effects of EA on depression and its mechanisms of action via IDO1 and the KP.

**Methods:**

An inflammation‐induced depression mouse model was created by injecting lipopolysaccharide (LPS) intraperitoneally. After the model was successfully established, the Hegu (L14) and Taichong (LR3) acupoints were treated with EA at a frequency of 15 Hz and an intensity of 0.3 mA for 45 min. A subset of mice received ketamine as an antidepressant intervention. The following assessments were performed: open‐field test (OFT), sucrose preference test (SPT), forced swim test (FST), and tail suspension test (TST). An enzyme‐linked immunosorbent assay (ELISA) was used to measure IDO1 levels and related inflammatory factors in brain tissue and peripheral blood. High‐performance liquid chromatography (HPLC) was used to measure KP metabolism‐related metabolite levels. Quantitative real‐time polymerase chain reaction (qRT‐PCR) was used to measure IDO1 and inflammation‐related factor levels.

**Results:**

LPS significantly induced depression‐like behavior in mice. Ketamine effectively alleviated this behavior, exerting an antidepressant effect and suppressing IDO1 expression, as well as the levels of inflammatory factors (IL‐1β, TNF‐α, and IL‐6). Following EA intervention, depression‐like behavior in mice with LPS‐induced depression was alleviated. EA effectively reduced peripheral and central inflammatory factor expression levels and suppressed IDO1 expression. EA alleviated depressive symptoms in mice by modulating the IDO1‐mediated KP.

**Conclusion:**

EA effectively reduced the expression of inflammatory factors, alleviated LPS‐induced depressive‐like behavior, and improved depressive symptoms by modulating the IDO1‐mediated KP.

## 1. Introduction

Depression is one of the most prevalent mental illnesses worldwide, causing health problems and socioeconomic burdens for many. Many factors contribute to depression, including family history, early‐life trauma and neglect, recent life stressors, and certain medications [1]. Additionally, stress resulting from other diseases (e.g., cancer), inflammation, oxidative stress, impaired immune function, and nervous system abnormalities can potentially trigger depression onset [2, 3]. Depression has many causes, and increasing evidence suggests that inflammation plays an important role in its development [4]. Pro‐inflammatory cytokines and markers of oxidative stress contribute to depression, and persistent inflammatory stimuli continuously exacerbate it [5]. The kynurenine (KYN) pathway (KP), an important regulatory pathway between inflammation and depression, has been shown to be strongly associated with inflammation and depression. The tryptophan (TRP) metabolic steps involved in the KP play an important role in the pathogenesis and treatment of depression [6, 7].

The lipopolysaccharide (LPS)‐induced model of inflammation‐associated depression has been widely used in many studies and is considered a relatively stable model [8]. Pro‐inflammatory cytokines produced by inflammation activate indoleamine 2,3‐dioxygenase (IDO), which converts TRP to KYN in the first rate‐limiting step of KP [6], reduces conversion of TRP to 5‐hydroxytryptamine (5‐HT) [9], and further metabolism of KYN to neurotoxic KYN metabolites, including quinolinic acid (QA), an N‐methyl‐D‐aspartate (NMDA) receptor agonist [10], increases peripheral and central glutamate (Glu) concentrations, further exacerbating depression [11].

Pharmacological modulation of KP metabolites (such as ketamine, memantine, and riluzole) can also alleviate depression to some extent [6]. 1‐Methyltryptophan (1‐MT), a pharmacological competitive antagonist of IDO, has been shown to ameliorate depression in mice by inhibiting IDO [12]. Ketamine, as a noncompetitive antagonist of NMDA receptors, is one of the drugs with better antidepressant effects and a faster onset of action [13]. Other studies have shown that ketamine has some anti‐inflammatory effects [14], but whether the antidepressant mechanism behind it is related to anti‐inflammation is not clear. Given that some of the antidepressants currently in use are expensive, and that low‐cost alternatives in the same class may be associated with various side effects, as well as issues such as delayed onset of action, incomplete efficacy, or adverse reactions, there is an urgent need to develop a safe, side‐effect‐free, and cost‐effective treatment for depression.

Electroacupuncture (EA) is a new form of treatment that combines traditional acupuncture with an electrical pulse therapy device and offers several advantages over traditional antidepressants, with good acceptability and fewer side effects [15, 16]. Our previous study showed that EA can alleviate LPS‐induced depression‐like behavior by inhibiting IDO‐regulated KP [17]; EA can regulate central nervous system plasticity by modulating PNN and its components in the extracellular matrix [18, 19]; simultaneously, EA can regulate central nervous system repair by inhibiting inflammasomes to modulate microglial polarization [20, 21]. Numerous studies have extensively demonstrated the anti‐inflammatory effects of EA [22–24]; however, there appears to be little research on the mechanism by which EA exerts its anti‐inflammatory effects to thereby prevent the exacerbation of depression. We therefore hypothesize that depression is partially caused by inflammation; if so, could suppressing inflammation directly influence the course of depression? Based on our previous findings, it remains unclear whether EA can treat depression by inhibiting IDO through its anti‐inflammatory effects, thereby affecting KP.

Our previous research demonstrated that 1‐MT inhibits IDO1 and alleviates depression; therefore, we used knockout (KO) mice to knock out IDO1 in order to investigate the critical role of IDO1 in inflammation‐induced depression [17]. Ketamine is used clinically as a fast‐acting antidepressant, but its use is often associated with side effects such as hallucinations, addiction, and nausea. Using ketamine as a positive drug control, we further explored the advantages of EA in the treatment of depression [25].

Therefore, this study utilized an LPS‐induced mouse model of depression to investigate the mechanism of action of EA in treating depression by measuring depression‐like behaviors, inflammatory cytokines, KP metabolites, and IDO levels, and to explore the advantages of EA over ketamine.

## 2. Materials and Methods

### 2.1. Animals

Healthy C57/BL6 male mice (8–10 weeks old) were purchased from the Experimental Animal Centre of Zhejiang University of Traditional Chinese Medicine (Approval Number 20210315‐15). IDO1 KO mice were purchased from The Jackson Laboratory (Strain #005867). Wild‐type (WT) mice and KO mice were randomly assigned to different groups (8 mice per group), namely, the phosphate buffered saline (PBS) group, LPS group, LPS + EA group, LPS + shamEA group, LPS + ketamine group, KO‐PBS group, KO‐LPS group, KO‐shamEA group, and KO‐EA group. For the randomization procedure, an operator unrelated to the experiment fitted all mice with ear tags, wrote a unique number on each slip of paper, and placed the slips into an opaque cardboard box. When assigning groups, a slip was drawn from the box, and the number on it was recorded to determine the group assignment. The WT and KO mice groups were randomized separately. We gave the mice at least 1 week to acclimatize to the environment before the experiments and kept them in a standard environment (room temperature 25 ± 2°C and 12:12 h of alternating light and darkness), where the animals had free access to food and water. The experimental animals were handled in accordance with the Guidelines on the Ethical Treatment of Laboratory Animals issued by the Ministry of Science and Technology of China in 2006, and in accordance with the animal experiment protocol approved by the Animal Ethics Committee of Zhejiang University of Traditional Chinese Medicine (ZSLL, 2017183).

### 2.2. LPS Induction Models and Drugs

LPS (L‐3129, serotype 0127:B8; Sigma) was diluted in sterile endotoxin‐free PBS solution. Intraperitoneal injection at a concentration of 0.83 mg/kg was used to study an inflammation‐induced depression model [26], a dose that has been shown to adequately and reliably induce acute illness behaviors and depressive‐like behaviors at the time points studied [27]. The control group (PBS group) was injected intraperitoneally with saline (5 mL/kg), and all the remaining groups were injected with LPS (Figure 1).

**Figure 1:**
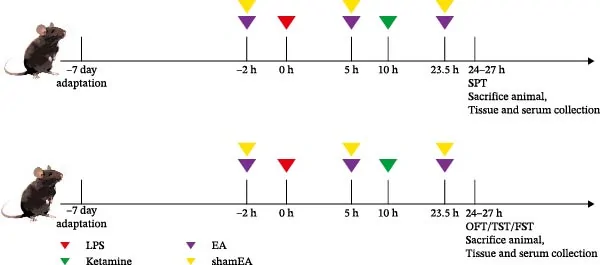
Timeline of experimental design. Mice were acclimatized for 7 days, and ketamine was administered 10 h after LPS injection; and EA and shamEA treatments were administered once 2 h before LPS injection and repeated 5 and 23.5 h after LPS injection; behavioral tests were performed 24–27 h after LPS. Each group contained four behavioral tests as follows: sucrose preference test (SPT), open field test (OFT), forced swimming test, and tail suspension test (TST).

Ketamine (2 mL: 50 mg, East China Pharmaceutical Co., Ltd., China) was administered intraperitoneally at a concentration of 10 mg/kg [13, 28] 10 h after LPS injection. The stock solution was diluted with saline at a ratio of 1:11.5 and used now.

### 2.3. EA Intervention

The selection of EA acupoints and frequencies was consistent with the results of previous studies [15, 17], the mice in the EA group received acupuncture and electrical stimulation at the Hegu (L14) and Taichong (LR3) acupoints for 45 min on an EA device (HANS‐200E; Huawei Co., Ltd., Beijing, China), and the diameters and lengths of the acupuncture needles used were 0.16 and 7 mm, respectively. The parameters used for EA were as follows: frequency: 15 HZ, intensity: 0.3 mA, and time: 45 min. EA treatments were performed 2 h before LPS injection, 5 h after LPS injection, and 23.5 h after LPS injection (Figure 1); meanwhile, the shamEA group used needles to puncture into the Hegu (L14) and Taichong (LR3) points without receiving electrical stimulation. The shamEA group refers to a group that uses standard acupuncture needles, with the same insertion depth and retention time as the EA group, but is distinguished from the EA group by the fact that the needles are connected only to the leads, and the EA device is turned off.

### 2.4. Behavioral Tests

All behavioral assessments and data analyses were conducted by two technicians who were not involved in this experiment. The open‐field test (OFT), FST, and tail suspension test (TST) behavioral tests were conducted on the same day, beginning with the OFT, followed by the TST, and concluding with the FST. The sucrose preference test (SPT) was conducted 2 h after the completion of these three tests. Behavioral tests for all groups were conducted in the same order and began at the same time each day. Additionally, the mice were given at least 1 h of rest between each behavioral test.

### 2.5. Body Weight Measurement

To observe the changes in the body of mice during LPS administration, we measured the body weight of each mouse at 2, 6, 10, and 24 h after LPS injection.

### 2.6. OFT

To eliminate nonspecific differences, each mouse in each group was subjected to a 5‐min OFT test to rule out differences [27]. In a black rectangular box with a square bottom (45 cm H × 45 cm L × 45 cm W) and nine equal‐sized areas (15 cm × 15 cm) were created. Mice in the central area of the mine were videotaped using a real‐time video tracking system (Ethovision, Nodus Information Technology) to track their movements and behavior.

### 2.7. SPT

Prior to the formal conduct of the experiment, mice were placed in a single cage in the field, and each mouse in each group was given 1% sucrose for 24 h. After a 6‐h water deprivation period, each mouse began a 3‐h test during which it was given one bottle of 1% sucrose solution and one bottle of water. At the 1.5‐h mark, the positions of the two bottles were switched to prevent lateral preference. The bottles were removed and weighed to determine fluid consumption. Sugar‐water preference index (SPI) = (sugar‐water intake/total intake) × 100%.

### 2.8. Forced Swimming Test

Each mouse was placed in a cylinder made of glass (21 cm high and 14.5 cm in diameter), and water at a temperature of 23 ± 1°C was added to a height of 15 cm to ensure that the mice were in a floating state. They were forced to perform a swimming test for 6 min, and the state when the mice were floating and stationary and could keep their noses out of the water was defined as immobile behavior, and the total immobility time of each mouse was measured. The water was changed once after each mouse completed the test to avoid interfering with the experiment. Mice that had completed the swim test were placed in heated cages for drying and returned to their original cages after 20 min.

### 2.9. TST

The tail of each mouse, which had been acclimatized for 1 h, was taped to a suspension rod in a black rectangular space (H 55 cm × L 15 cm × W 11.5 cm), with a black partition separating neighboring mice, and the immobilization time of the mice was recorded and analyzed by video recording for a period of 6 min.

### 2.10. Biochemical Analysis

#### 2.10.1. Determination of IDO1 Levels in Brain Tissue and Serum

Brain tissue and serum were collected to analyze IDO1 levels. The serum was separated from naturally clotting blood and then centrifuged at 3000 rpm for 10 min. An appropriate volume of PBS (1 mL/10 mg) was added to the brain tissue. After homogenization and centrifugation, samples were centrifuged at 4°C for 20 min (3000 rpm), and the supernatant was collected for further analysis. Sample IDO1 levels were determined according to the protocol provided in the enzyme‐linked immunosorbent assay (ELISA) kit (JL47052) (Jianglai, Shanghai, China). The corresponding absorbance (OD) was measured at 450 nm for each sample.

#### 2.10.2. Determination of Inflammatory Cytokine Levels in Brain Tissue and Serum

Serum and brain tissue (prefrontal cortex [PFC] and hippocampus [HIP]) of mice were collected for the inflammatory cytokine assay. Inflammatory cytokine levels were measured according to the instructions of IL‐6 (JL20268), IL‐1β (JL JL18442), and TNF‐α (JL10484) ELISA kits (Jianglai, Shanghai, China). The samples were added to three duplicate wells, and the analysis was repeated three times. The absorbance (OD) of each sample was measured at 450 nm.

#### 2.10.3. Measurement of Glu and KP Metabolites in Brain Tissue and Serum

Liquid chromatography separation was performed according to the method described in previous studies [29]. Samples were analyzed using an Agilent high‐performance liquid chromatography (HPLC)‐1100 system (Agilent Technologies, USA). A Diamonsil AAA column (4.6 mm × 250 mm, particle size 5 μm) was used. The flow rate was 1 mL/min, the injection volume was 20 μL, and the mobile phase A consisted of acetonitrile/methanol (90/10); mobile phase B consisted of 0.02 mol/L sodium dihydrogen phosphate and disodium hydrogen phosphate using a gradient elution method. The concentration of the sample was determined using the external standard method. The standard was serially diluted with the mobile phase to create six concentration gradients; three samples were injected at each concentration point, and the peak areas were recorded. A linear curve was plotted with the peak area on the *y*‐axis and standard concentration on the *x*‐axis; when *R* ^2^ > 0.999, the linear fit was considered good. Glu, TRP, and KYN were detected using a UV detector, while QA was detected using a fluorescence detector.

#### 2.10.4. Quantitative Real‐Time Polymerase Chain Reaction (qRT‐PCR)

Total RNA was extracted from rapidly decapitated mouse PFC and HIP using TRizol (Invitrogen, Carlsbad, USA). Prior to RNA reverse transcription, the purity of RNA is assessed. Samples with an A260/A280 ratio between 1.90 and 2.10 and an A260/A230 ratio greater than 2.0 are included in the analysis; samples that do not meet these requirements are excluded. After completing reverse transcription according to the instructions in the reverse transcriptase kit (TaKaRa), the primers were analyzed using qPCR melting curves to ensure that each primer produced only a single amplification product. At the same time, the cDNA template was serially diluted in a 5‐fold gradient, and a standard curve was generated using qPCR. The amplification efficiency was calculated using the formula *E* = 10^(-1/k)^ – 1; an amplification efficiency between 90% and 110% was considered optimal. Real‐time PCR detection of the target gene was performed using TB Green (TaKaRa) on a Roche PCR system (PCR480). Each sample was added to three replicate wells to perform the analysis three times, and the results were averaged. Gene expression was calculated using the 2^-ΔΔCT^ method, and the final data were normalized by beta actin. The primer sequences are shown in Table 1.

**Table 1:** Sequences of the primers used for qRT‐PCR.

Primers	Forward	Reverse	Amplicon size (bp)
Actin	CACCCGCGAGTACAACCTTC	CCCATACCCACCATCACACC	207
IL‐6	AGAGACTTCCAGCCAGTTGC	CTGGTCTGTTGTGGGTGGTA	115
IL‐1β	CAACTGTTCCTGAACTCAACTG	GAAGGAAAAGAAGGTGCTCATG	145
TNF‐α	GATCGGTCCCAACAAGGAGG	GCTTGGTGGTTTGCTACGAC	138
IDO1	CGAGAACATGGACATTCTGTTC	TTTCCAATGCTTTCAGGTCTTG	158

### 2.11. Statistical Analyses

GraphPad Prism 8 (GraphPad Software, San Diego, CA, USA) was used for statistical analysis. The normality of the continuous variables was assessed using the Shapiro–Wilk test. For data that follow a normal distribution and satisfy the assumption of homogeneity of variances, the Stewart *t*‐test (two‐tailed) was used for comparisons between two groups, while one‐way analysis of variance (ANOVA) was used for comparisons among multiple groups, followed by Tukey’s post hoc test. For data that do not follow a normal distribution or satisfy the assumption of homogeneity of variances, the Mann–Whitney *U* test and the Kruskal–Wallis *H* test were used for comparisons between two groups and among multiple groups, respectively. A two‐way ANOVA was used to analyze the data, which involved two variables. When an interaction between group and time was observed, a simple effects analysis was performed, followed by Tukey’s post hoc test for adjustment; when no interaction between group and time was observed, a main effects analysis was performed, followed by Tukey’s post hoc test. All results are expressed as the mean ± standard error of the mean (SEM). A *p*  < 0.05 is considered statistically significant.

## 3. Results

### 3.1. Ketamine Attenuates LPS‐Induced Depression‐Like Behavior

Disease‐ or depression‐like behavior was induced via an intraperitoneal injection of either LPS (0.83 mg/kg) or saline. Prior to euthanasia, the mice were weighed, and the appropriate behavioral tests were performed. Then, the mouse serum or brain tissue was collected for analysis. We measured the change in body weight 24 h after LPS injection (*p* < 0.0001) (Figure 2A) and the change in sucrose preference (*p* = 0.0114) (Figure 2B). Each mouse underwent a 5‐min OFT to evaluate the motor function; no significant differences were observed among the tested mice (Figure 2C). Unlike mice in the LPS group, ketamine effectively increased the activity time of mice in the central region (*p* = 0.0035) (Figure 2D). Additionally, ketamine reduced the immobility time of mice in the FST and TST (FST: *p* = 0.0016, TST: *p* = 0.0124) (Figure 2E,F).

**Figure 2:**
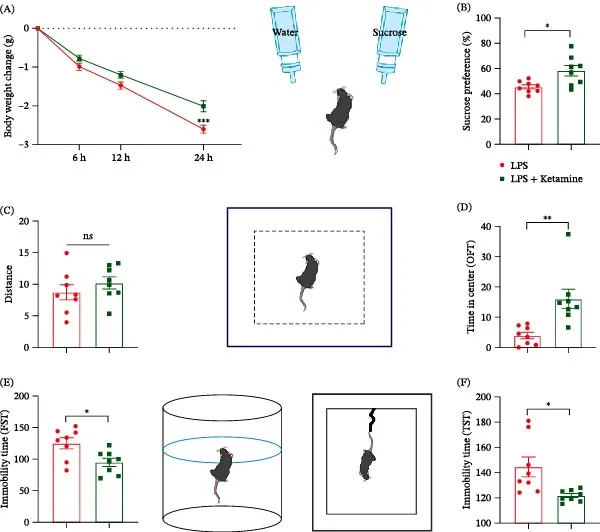
Ketamine attenuated LPS‐induced depression‐like behavior. (A) Body weight changes in both LPS and LPS + ketamine groups in 6, 12, and 24 h. (B) Effect of ketamine on sucrose preference in LPS‐stressed mice. (C) Total distance moved by LPS and LPS + ketamine groups in the mine field experiment. (D) Effect of ketamine administration on the residence time in the central region of LPS‐stressed mice. (E) Statistical graphs of immobility time in FST for both LPS and LPS + ketamine groups of mice. (F) Statistical graphs of immobility time in TST for both LPS and LPS + ketamine groups of mice. Results are expressed as SEM ± mean (*n* = 7–8 per group;  ^∗^ *p*  < 0.05,  ^∗∗^ *p*  < 0.01).

### 3.2. Ketamine Inhibits IDO1 Expression and Attenuates Inflammatory Factors in Periphery and Center

To examine the effects of ketamine on the expression of inflammatory factors and IDO1 in central brain tissue and peripheral blood, we euthanized the mice 27 h after LPS injection and rapidly extracted serum and brain tissue (PFC and HIP) for assay analysis [30, 31].

As shown by the ELISA results in Figure 3, ketamine significantly reduced the protein expression levels of inflammatory factors IL‐6, IL‐1β, and TNF‐α in brain (IL‐6: *p* < 0.0001, IL‐1β: *p* = 0.0001, TNF‐α: *p* < 0.0001) and serum (IL‐6: *p* < 0.0001, IL‐1β: *p* = 0.0017, TNF‐α: *p* = 0.0007) (Figure 3A–C,E–G). However, qPCR results showed that ketamine did not reduce the mRNA expression of IL‐6, IL‐1β, and TNF‐α in brain tissues (PFC and HIP) (Figure 3I–K), a result that is consistent with a previous report [32] that ketamine use during acute inflammation increases IL‐6, IL‐1β and TNF‐α mRNA levels.

**Figure 3:**
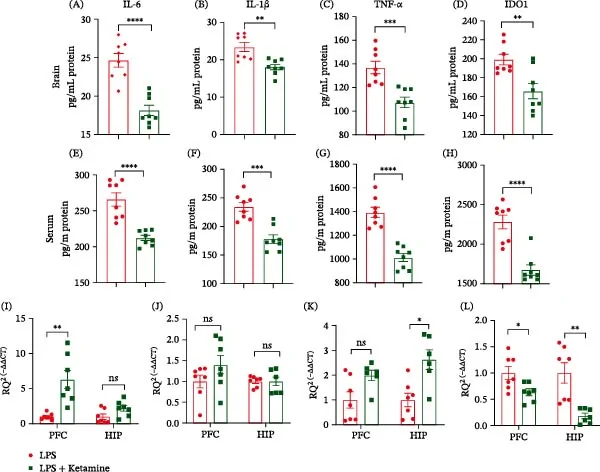
Ketamine inhibited the expression of inflammatory factor proteins and IDO1. (A–D) Protein levels of IL‐6, IL‐1β, TNF‐α, and IDO1 in brain tissues determined by ELISA. (E–H) Protein levels of IL‐6, IL‐1β, TNF‐α, and IDO1 in serum determined by ELISA. (I–L) Transcript levels of IL‐6, IL‐1β, TNF‐α, and IDO1 in the PFC and HIP regions of the mouse brain determined by PCR. Results are expressed as SEM ± mean (*n* = 7–8 per group;  ^∗^ *p*  < 0.05,  ^∗∗^ *p*  < 0.01,  ^∗∗∗^ *p*  < 0.001,  ^∗∗∗∗^ *p*  < 0.0001).

Meanwhile, we tested the protein and gene levels of IDO1, and the protein expression of IDO1 in the peripheral and central areas was reduced by ketamine compared with the LPS group (brain: *p* < 0.0001, serum: *p* = 0.0042) (Figure 3D,H). qPCR results were consistent with the ELISA results, and ketamine effectively reduced the mRNA expression of IDO1 in brain tissue (PFC: *p* = 0.0215, HIP: *p* = 0.0016) (Figure 3L).

### 3.3. Ketamine Inhibits IDO1 and Modulates KP Metabolites to Relieve Depression

To confirm whether ketamine achieves antidepressant effects by inhibiting IDO1 and thereby regulating KP, we measured the concentrations of TRP, KYN, QA, and Glu in peripheral and central areas. We found that ketamine significantly reduced KYN levels and KYN/TRP ratios in serum and brain tissues (Figure 4A–D) [33]. Correspondingly, we observed that ketamine decreased QA concentrations in serum (*p* = 0.0177) and brain tissues (*p* = 0.0101) (Figure 4E,G). Glu, as an excitatory neurotransmitter, can produce some neurotoxicity and lead to depression. While the use of ketamine significantly reduced Glu levels in the brain tissue (Figure 4F), there appeared to be no change in serum (Figure 4H).

**Figure 4:**
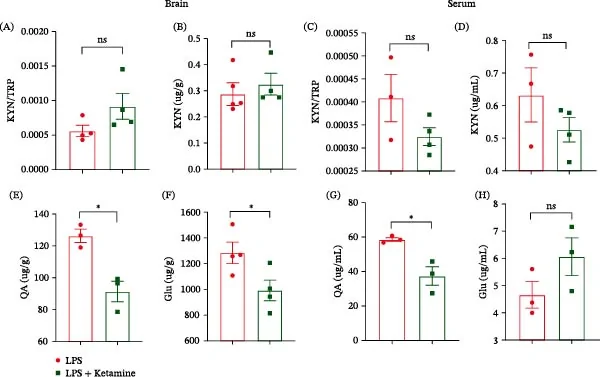
Ketamine reduced the metabolic levels of QA and Glu. (A, B, E, F) Metabolic levels of KYN/TRP (A), KYN (B), QA (E), and Glu (F) determined by HPLC in mouse brain tissues collected after behavioral testing. (C, D, G, H) Metabolic levels of KYN/TRP (C), KYN (D), QA (G), and Glu (H) determined by HPLC in mouse serum collected after behavioral testing metabolic levels. Results are expressed as SEM ± mean (*n* = 3–4 per group;  ^∗^ *p*  < 0.05).

### 3.4. EA Alleviates LPS‐Induced Depressive‐Like Behavior

In previous studies, we have shown that EA can alleviate depressive‐like behaviors by modulating KP [17], but whether EA can alleviate depression‐like behavior through anti‐inflammatory modulation of IDO‐mediated KP remains to be determined. To this end, we used targeted KO of the IDO1 gene to successfully construct IDO1 KO mice (Figure 5A), which are characterized by the absence of the enzyme produced by the IDO1 gene in the body (Figure S1A,B), thus blocking the pathway that affects the KY pathway through IDO1. We measured the body weight changes of WT and KO mice over 24 h after LPS injection, and the LPS‐induced body weight reduction in KO mice was attenuated by EA, and WT mice showed the same result, that is, EA attenuated the LPS‐induced body weight reduction to some extent (interaction: F_(21, 224)_ = 5.730, *p* < 0.0001) (Figure 5B). In OFT, WT mice had a significant reduction in activity time of their central region by LPS injection, which was significantly reversed by EA (WT‐LPS/WT‐EA: *p* = 0.0004); however, after KO‐IDO1, LPS injection failed to induce a significant depressive‐like behavior in KO mice (WT‐LPS/KO‐LPS: *p* = 0.0055) (Figure 5F), and there was no significant difference between the groups of mice. There was no difference in the motor function between WT and KO mice (Figure 5E).

**Figure 5:**
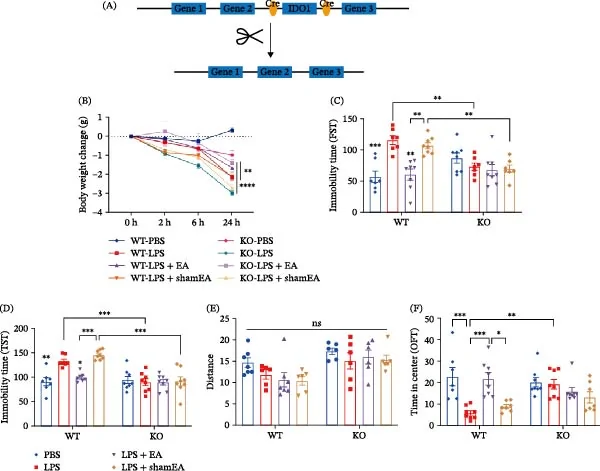
EA alleviates LPS‐induced depressive‐like behavior through IDO1. (A) Simple schematic diagram of IDO1 knockout. (B) Body weight changes of WT and KO mice at 2, 6, and 24 h. (C, D) Statistical graphs of immobility time in FST, TST for each of the four groups of mice in WT and KO. (E) Total distance crawled by WT and KO mice in OFT. (F) Activity time of the central region of the OFT for WT and KO mice. Results are expressed as SEM ± mean (*n* = 7–8 per group;  ^∗^ *p*  < 0.05,  ^∗∗^ *p*  < 0.01,  ^∗∗∗^ *p*  < 0.001,  ^∗∗∗∗^ *p*  < 0.0001).

In the FST, WT mice were injected with LPS to increase immobility time, which was significantly reversed in the EA group (WT‐LPS/WT‐EA: *p* < 0.0001), with a significant decrease in immobility time (Figure 5C). However, the depression‐like behavior induced by LPS injection did not significantly increase the immobility time in the KO groups, and the immobility time was significantly increased in the WT‐LPS group compared with the KO‐LPS group, indicating that the depression‐like behavior in the KO‐LPS group mice was significantly decreased (WT‐LPS/KO‐LPS: *p* = 0.0070) (Figure 5C), this result was also applicable to the sham‐EA group. There was no significant difference between the groups of KO mice. In the TST, the results were consistent with those in the FST (Figure 5D).

### 3.5. EA Blocks Inflammatory Factors and Inhibits IDO1 Expression

To investigate whether EA could achieve antidepressant effects by inhibiting IDO regulation of KP or affect IDO by modulating inflammation, we examined the protein levels of IL‐6, IL‐1β, and TNF‐α, the relevant inflammatory factors, in WT and KO mice, both peripherally and centrally. In WT mice, the protein expression levels of IL‐6, IL‐1β, and TNF‐α in serum and brain tissues significantly increased after LPS injection, whereas EA significantly inhibited the protein expression of inflammatory factors (Figure 6A–C,E,F); in KO mice (KO‐EA/KO‐LPS: brain: IL‐6: *p* = 0.0245, IL‐1β: *p* = 0.0494, TNF‐α: *p* = 0.0054; serum: IL‐6: *p* = 0.0419, IL‐1β: *p* = 0.0300, TNF‐α: *p* = 0.0302), the results were consistent with those of WT mice (Figure 7A–F).

**Figure 6:**
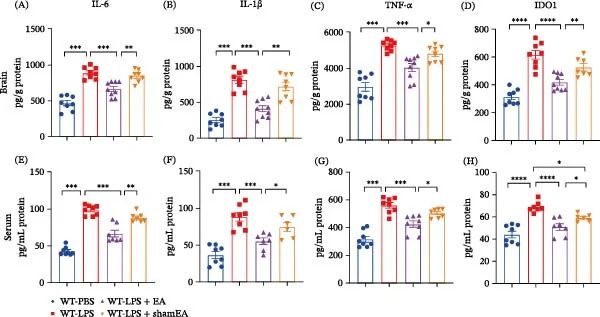
EA inhibited protein levels of inflammatory factors and IDO1 in brain and serum of WT mice. (A–D) Protein levels of IL‐6, IL‐1β, TNF‐α, and IDO1 in brain tissues of WT mice measured by ELISA. (E–H) Protein levels of IL‐6, IL‐1β, TNF‐α and IDO1 in serum of WT mice measured by ELISA. Results are expressed as SEM ± mean (*n* = 7–8 per group;  ^∗^ *p*  < 0.05,  ^∗∗^ *p*  < 0.01,  ^∗∗∗^ *p*  < 0.001,  ^∗∗∗∗^ *p*  < 0.0001).

**Figure 7:**
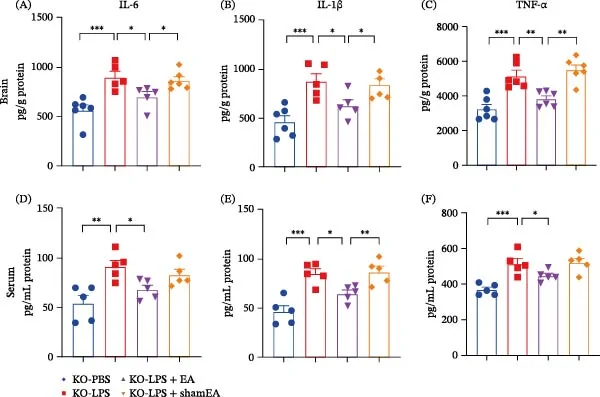
EA inhibited protein levels of inflammatory factors in brain and serum of KO mice. (A–C) Protein levels of IL‐6, IL‐1β, and TNF‐α in brain tissues of KO mice measured by ELISA. (D–F) Protein levels of IL‐6, IL‐1β, and TNF‐α in serum of KO mice measured by ELISA. Results are expressed as SEM ± mean (*n* = 8;  ^∗^ *p* < 0.05,  ^∗∗^ *p* < 0.01,  ^∗∗∗^ *p* < 0.001).

The inhibitory effect of EA on IDO was confirmed in a previous study [17], which we repeated here, and the results in WT mice were consistent with the previous study (Figure 6D,H). However, in KO mice, the inhibition of IDO was at a lower level and not significantly different between each group due to the IDO gene KO (results not shown).

### 3.6. EA May Alleviate Depression‐Like Behavior by Modulating IDO1‐Mediated KP

In contrast to ketamine, which fails to suppress inflammation at the transcriptional level, EA not only reduced the mRNA expression of IL‐6, IL‐1β, TNF‐α, and IDO1 in the PFC and HIP of WT mice (Figure 8A–H), but also significantly reduced the mRNA expression of these inflammatory factors in KO mice (PFC: IL‐6: *p* = 0.0003, IL‐1β: *p* = 0.0002, TNF‐α: *p* = 0.0435; HIP: IL‐6: *p* = 0.0018, IL‐1β: *p* = 0.0076, TNF‐α: *p* = 0.0466) (Figure 9A–F).

**Figure 8:**
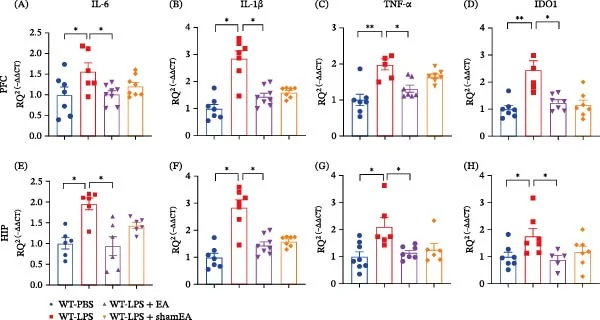
EA inhibits RNA expression of inflammatory factors and IDO1 in the brain of WT mice. (A–D) Transcript levels of IL‐6, IL‐1β, TNF‐α, and IDO1 in PFC of WT mice were determined by PCR. (E–H) Transcript levels of IL‐6, IL‐1β, TNF‐α, and IDO1 in HIP of WT mice were determined by PCR. Results are expressed as SEM ± mean (*n* = 8;  ^∗^ *p* < 0.05,  ^∗∗^ *p* < 0.01).

**Figure 9:**
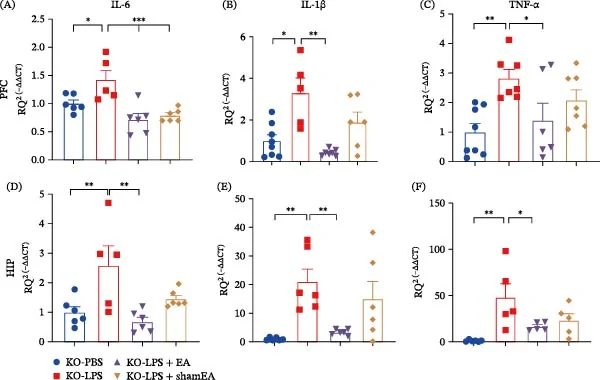
EA inhibits RNA expression of inflammatory factors in the brain of KO mice. (A–C) Transcript levels of IL‐6, IL‐1β, and TNF‐α in PFC of WT mice were determined by PCR. (D–F) Transcript levels of IL‐6, IL‐1β, and TNF‐α in HIP of WT mice were determined by PCR. Results are expressed as SEM ± mean (*n* = 8;  ^∗^ *p* < 0.05,  ^∗∗^ *p* < 0.01,  ^∗∗∗^ *p* < 0.001).

After assessing the concentrations of KYN, QA, and Glu in the peripheral and central nervous systems, we found that EA intervention reduced the LPS‐induced increases in KYN and QA levels (Figure S2B,D,E,G), lowered the KYN/TRP ratio (Figure S2A,C), and decreased Glu concentrations in the brain tissue (Figure S2F), although the changes in serum Glu were less pronounced (Figure S2H). When we compared WT with KO mice, the inflammatory pathway of IDO1 affecting KP was blocked by IDO1 gene deletion in KO mice, and the concentrations of KYN, QA, and Glu in brain tissue and serum of KO mice were unchanged by LPS application (Figure S3B,D–H), whereas an increase was observed in WT mice (Figure S3B,D–H); the ratio of KYN/TRP did not change significantly between the two groups of KO mice (Figure S3A,C), whereas it was significantly elevated in the WT‐LPS group compared with the WT‐PBS group and differed in brain tissue.

## 4. Discussion

A growing body of evidence shows that inflammation and depression are closely linked. The reciprocal cycle in which inflammation promotes depression and depression promotes inflammation poses a significant risk to physical and mental health [34]. Pro‐inflammatory cytokines, which are released by inflammation, alter the production, metabolism, and transport of neurotransmitters, such as Glu, dopamine, and serotonin [35]. These cytokines also damage neurons in the brain and alter emotional activity, leading to varying degrees of depression [36, 37].

Researchers have found that inflammation activates IDO, which slows down 5‐HT production and increases KYN levels. This ultimately affects Glu receptors in the brain and promotes depression [38]. This process is primarily accomplished by KP. Several IDO inhibitors and drugs that modulate KP metabolites are in development and undergoing testing. The best‐known IDO inhibitor, 1‐MT, and the NMDA receptor antagonist ketamine, have been extensively researched. Ketamine is a well‐recognized, rapid‐acting antidepressant that has been used in various animal models and clinical trials [13, 39, 40]. Some studies have confirmed that ketamine has anti‐inflammatory effects and inhibits NMDA receptor activity [41] by noncompetitive antagonism of the NMDA receptor [42], which has the opposite effect of the QA produced by inflammation‐activated, IDO‐promoted KP metabolism. Ketamine reduces Glu release to alleviate depression.

Our previous research indicates that EA intervention may have certain antidepressant effects on inflammatory depression‐like behavior [17, 43], with effects that are similar to those of ketamine. Because the antidepressant effects of ketamine have been extensively studied in clinical and animal trials, and because its effects are stable, we used ketamine as a positive antidepressant control to further investigate the antidepressant mechanisms of EA. Our study revealed that ketamine has a significant antidepressant effect on acute LPS‐induced depressive‐like behavior and an anti‐inflammatory effect on pre‐existing inflammation (peripheral or central). However, it does not inhibit inflammatory factors at the transcriptional level, which is consistent with previous reports [32]. Ketamine effectively inhibited IDO1 upregulation at both the protein and transcriptional levels. Ketamine also reduced Glu concentrations and lowered the KYN/TRP ratio peripherally and centrally. This demonstrates that ketamine can affect the KP to alleviate depression by modulating IDO. Although ketamine has been used in some clinical treatments, it has significant side effects, including nausea, an accelerated heart rate, dissociation, headaches, and elevated blood pressure [44]. Repeated use can lead to decreased antidepressant effects [45] and addiction [44].

Due to the numerous side effects and issues associated with the clinical use of ketamine, we selected the EA intervention method, which has been reported in many studies to be relatively safe, simple, and effective. The safety and efficacy of EA have been demonstrated in most studies [46–50]. Some studies have confirmed that EA can alleviate depression by preventing astrocyte atrophy [51], enhance synaptic transmission in the ventromedial PFC (vmPFC) to achieve antidepressant effects [52], reduce pain caused by chronic inflammation [53], and be used as a complementary therapy for antidepressants [54]. While the antidepressant effects of EA have been elaborated on in various studies, most have only addressed the cellular level or certain factors and have not fully elucidated the mechanistic pathways of EA’s antidepressant effects. To elucidate the specific mechanisms underlying EA’s antidepressant effects related to this pathway, we focused on the LPS‐induced depression model. Through comparative experiments on the antidepressant effects of EA and ketamine, as well as studies on inflammation, IDO, and KP metabolites in IDO‐KO and WT mice, we confirmed that EA’s antidepressant mechanism involves modulating IDO to influence KP and its metabolites. Furthermore, EA’s effects on inflammatory factors and IDO1 can induce changes at the DNA level. This represents a clear distinction from ketamine, and these findings constitute the innovative aspects of this study.

In our experiments, we prescreened EA sites and frequencies and used acupuncture at the Hegu (LI4) point, located at the midpoint of the second metacarpal bone on the radial side, and the Taichong (LR3) point, located at the lateral end of the second toe [15, 17]. These points have been shown to alleviate depressive‐like behavior induced by chronic unpredictable mild stress (CUMS) [17, 43] and activate brain regions involved in emotional cognition [55].

Our findings suggest that EA decreases the levels of IL‐6, IL‐1β, TNF‐α, and IDO1 in the brain tissue and peripheral blood of WT mice and reduces inflammatory factor and IDO1 levels at the transcriptional level. These effects may be related to the mechanisms of the EA. Numerous studies have shown that EA exerts anti‐inflammatory effects by modulating neural circuits [56]. Afferent signals generated by EA converge in the central nervous system and affect regions such as the nucleus of the solitary tract in the medulla oblongata, the hypothalamus, and the midbrain. These regions integrate the afferent signals and transmit them to structures such as the HIP, PFC, and amygdala. This process regulates immune homeostasis and central inflammation [57–59]. EA has also been shown to modulate the balance between pro‐inflammatory and anti‐inflammatory signaling pathways. One key pathway is the Toll‐like receptor 4 (TLR4)/nuclear factor kappa‐B (NF‐κB) pathway, which plays a crucial role in central nervous system inflammation and glial cell activation. Previous studies have confirmed that EA downregulates TLR4 and NF‐κB, significantly reducing the transcription of pro‐inflammatory cytokines, such as IL‐1β, IL‐6, and TNF‐α [60–64].

Given IDO1’s important role in TRP metabolism, we investigated the mechanisms of EA intervention for depression with a focus on inflammation and TRP metabolites. We also knocked down the IDO1 gene. As discussed in the Results section, the presence of the IDO1 gene is crucial for LPS‐mediated, inflammation‐induced depression. Additionally, the antidepressant effect of EA may be mediated through IDO1. When we compared WT and IDO1 KO mice, we found that the KP metabolites in the KO PBS group were higher than in the WT PBS group. While there were no significant differences between the two groups, previous literature suggests that, in certain cases of gene deletion, genes within the same subtype may exhibit a compensatory behavior that exerts additional effects. Therefore, we propose the following hypothesis: deletion of the IDO1 gene leads to compensatory changes in the IDO2 gene, which alters the KP metabolic pathway [65]. Since this hypothesis has not been experimentally verified, we may explore it in greater depth in future studies. Given that no significant changes in depression‐like behavior were observed in behavioral experiments with KO mice, we have reason to believe that these behavioral changes remain within the normal range.

In a study of inflammatory factors, deletion of the IDO1 gene was found not to alter the level of inflammation induced by LPS. Furthermore, inflammatory factor proteins remained elevated in the brain tissue and peripheral serum of KO mice, and there was a similar increase in transcriptional levels. These results suggest that the anti‐inflammatory effect of EA is not restricted to the IDO1 gene and that IDO1 deletion only restricts the pathway of inflammation‐induced depression through the KP. Further experiments are needed to determine whether EA can truly alleviate depression through anti‐inflammation. However, it is certain that EA possesses certain antidepressant effects and exhibits some degree of overlap with ketamine in regulating IDO1 and KP levels. Compared with many pharmacological interventions, EA has been reported to have a good safety profile; compared to ketamine, its anti‐inflammatory effects appear to have a broader scope.

The LPS‐induced acute inflammatory depression model lacks the chronic characteristics and manifestations commonly seen in major depressive disorder (MDD). Due to the model’s limitations, however, we are currently unable to determine whether EA has the same therapeutic effect on chronic depression or MDD. The results of our study suggest that inflammation located upstream of IDO1 is a key precursor to acute depression and that EA alleviates depression through IDO1 and the KP. However, it remains unclear whether the anti‐inflammatory effects of EA directly alleviate depression, which requires further investigation. Combined with the results of our previous studies, we have only examined the relationship between IDO and KP in inflammation within the CUMS [43], but we have not yet confirmed the therapeutic effects and mechanisms of EA for chronic depression. We have found that EA effectively alleviates inflammation‐induced depression and exerts dual anti‐inflammatory and antidepressant effects. To investigate the antidepressant mechanisms of EA and ketamine, we will explore the IDO1 downstream signaling pathways, such as PI3K/Akt and NF‐κB, in future studies. We will also quantify KP metabolites (e.g., KYN and aminotransferase) more frequently to better understand the effects of EA on this pathway. Since depression involves cognitive domains, we plan to conduct additional novel studies by testing the cognitive abilities of LPS‐induced depressed mice using the Y‐maze or the Morris water maze. Additionally, we will use flow cytometry to analyze changes in immune cell populations (e.g., microglia, T cells, and monocytes) in the brain and peripheral blood after EA or ketamine treatment. This will help us better define the effects of EA or ketamine on inflammation. This exploratory study was conducted exclusively on male mice and did not examine gender differences. If conditions permit, we will conduct further exploratory studies on mice of different genders in future research. Based on the results of our previous study, the relationship between IDO and KP remains valid in the CUMS mouse model of depression. Future studies will use the CUMS model or other chronic stress models and incorporate ketamine and EA interventions to validate the mechanisms underlying their antidepressant effects. In chronic stress models, inflammation is likely chronic, which differs significantly from acute inflammation currently under study. Therefore, we will use techniques such as immunofluorescence, Western blotting, and calcium imaging to analyze mouse models of acute and chronic depression. If necessary, we will conduct additional electrophysiological analyses. Since follow‐up studies are time‐consuming and unavailable in the short term, we have published only the preliminary results of this study in the hope of providing valuable arguments for the treatment of depression. Our findings offer potential theoretical and empirical support for the use of EA in treating depression and other mental disorders.

In conclusion, our findings demonstrate that EA modulates KP via IDO1 to alleviate depression caused by LPS‐induced acute inflammation. EA exerts a dual effect by reducing inflammation and regulating Glu metabolism. We have also identified the important role of IDO1 in inflammatory depression. However, this study has several limitations. First, it was conducted exclusively in male mice and did not account for sex differences. Second, there are limitations regarding the exploration of IDO1’s downstream pathways. These issues require further investigation. Nevertheless, EA has already demonstrated therapeutic effects in treating inflammatory depression. This may partially explain the phenomenon and provide a basis for EA to become a new depression therapy.

## Author Contributions

Bowen Chen and Xingying Wu contributed to this work. Qin Guo and Ruijie Ma designed this study. Bowen Chen, Xingying Wu, Xihan Ying, Shuo jiang, and Zhong Di analyzed the data. Bowen Chen, Yi Chen, and Jieqi Zhang wrote the manuscript. Xingying Wu, Yi Huang, and Hongen Chang participated in the revision of the article. Qin Guo and Ruijie Ma validated the manuscript.

## Funding

This study was supported by the National Natural Science Foundation of China (Grants 82004463 and 82174487) and the Zhejiang Traditional Chinese Medicine Science and Technology Plan Project (Grant 2023ZL475).

## Disclosure

All authors had read and approved the final version of the manuscript.

## Ethics Statement

The animal study was reviewed and approved by the Laboratory Animal Center of Zhejiang Chinese Medicine University.

## Conflicts of Interest

The authors declare no conflicts of interest.

## Supporting Information

Additional supporting information can be found online in the Supporting Information section.

## Supporting information


**Supporting Information** In Supporting Information, we present the PCR results of KO‐IDO1 and the rest of the sections with high‐performance liquid chromatography results, which illustrate that EA can be regulated through IDO1. Figure S1: No RNA expressing IDO1 in the brain of KO mice after knockdown of IDO1 gene. Figure S2: EA regulates the concentration levels of KYN, QA, and Glu. Figure S3: Knockdown of IDO1 gene reverses LPS‐induced increase in KP metabolites.

## Data Availability

Data are available upon request from the authors.
