# Supplementary material for: Electroacupuncture May Alleviate Depression and Exerts Anti‐Inflammatory Effects Through IDO1 Modulation of the Kynurenine Pathway
Source: Neural Plast. 2026 Nov 1;2026:6435038. doi: 10.1155/np/6435038 (PMC13725182; doi:10.1155/np/6435038)
Supplement: Supplementary file 1 [file NP-2026-6435038-s001.docx]

**Abstract**

In the Supplement, we present the PCR results of KO-IDO1 and the rest of the sections with high-performance liquid chromatography results, which illustrate that EA can be regulated through IDO1.

**Supplementary Figure 1 No RNA expressing IDO1 in the brain of KO mice after knockdown of IDO1 gene**

A-B: PCR detection of IDO1 RNA expression in the prefrontal cortex (PFC) and hippocampus (HIP) of KO mice.

**Supplementary Figure 2 EA regulates the concentration levels of KYN, QA and Glu**

A-B, E-F: HPLC detection of KYN/TRP ratio, KYN, QA and Glu concentrations in brain tissues of WT mice.C-D, G-H: HPLC detection of KYN/TRP ratio, KYN, QA and Glu concentrations in serum of WT mice. Results are expressed as SEM±mean (n=4. **p*<0.05, ***p*<0.01, ****p*<0.001).

**Supplementary Figure 3 Knockdown of IDO1 gene reverses LPS-induced increase in KP metabolites**

A-D: KYN/TRP ratio in brain tissue and serum. E-H: Concentration levels of KYN, QA and Glu in brain tissue and serum measured by HPLC in PBS and LPS groups of WT and KO mice. Results are expressed as SEM ± mean (n=4. **p*<0.05, ***p*<0.01).
